# Frustration and ennui among Amazon MTurk workers

**DOI:** 10.3758/s13428-022-01955-9

**Published:** 2022-08-26

**Authors:** Craig Fowler, Jian Jiao, Margaret Pitts

**Affiliations:** 1 School of Communication, Journalism, & Marketing, Massey University, Private Bag 102904, North Shore, Auckland, 0745 New Zealand; 2 Department of Communication, University of Arizona, Communication Building #222, 1103 E. University Blvd, Tucson, AZ 85721-0025 USA

**Keywords:** Crowdsourcing, Ethics, Digital methods, Internet, Job satisfaction, Online research, Participants

## Abstract

Academics are increasingly turning to crowdsourcing platforms to recruit research participants. Their endeavors have benefited from a proliferation of studies attesting to the quality of crowdsourced data or offering guidance on managing specific challenges associated with doing crowdsourced research. Thus far, however, relatively little is known about what it is like to be a participant in crowdsourced research. Our analysis of almost 1400 free-text responses provides insight into the frustrations encountered by workers on one widely used crowdsourcing site: Amazon’s MTurk. Some of these frustrations stem from inherent limitations of the MTurk platform and cannot easily be addressed by researchers. Many others, however, concern factors that are directly controllable by researchers and that may also be relevant for researchers using other crowdsourcing platforms such as Prolific or CrowdFlower. Based on participants’ accounts of their experiences as crowdsource workers, we offer recommendations researchers might consider as they seek to design online studies that demonstrate consideration for respondents and respect for their time, effort, and dignity.

Not long ago, Buhrmester et al. (2018) remarked on how rapidly Amazon Mechanical Turk (MTurk) went from being virtually unheard of to being ubiquitous across the social sciences. In 2009, for instance, just a handful of papers using MTurk were published in social science journals with impact factors of 2.5 or greater. This number had increased to almost 50 by 2011 and to just shy of 550 papers by 2015 (Chandler & Shapiro, 2016).

Political scientists were quick to show their enthusiasm for the possibilities afforded by MTurk (Christenson & Glick, 2013), as were scholars in psychology. As recently as 2012, for instance, fewer than 10% of papers published in seven top psychology journals reported studies using MTurk. In five of the same seven journals, however, the proportion of published studies using MTurk was at least 24% by 2017, and in two specialist social psychology journals, the figure exceeded 40% (Stewart et al., 2017; Zhou & Fishbach, 2016). A comparable proportion of studies (43%) published in the *Journal of Consumer Research* between June 2015 and April 2016 drew on MTurk data (Goodman & Paolacci, 2017), suggesting that some business-focused disciplines have also embraced the use of crowdsourced data. The communication discipline was one of the “later entrants to the crowdsourcing arena” (Sheehan, 2018, pp. 140–141), but many communication and media scholars have now joined colleagues in other social science fields in using this approach to data collection. For instance, Stansberry (2020) reviewed articles published in *Public Relations Review* during 2018 and found that 42% of articles employing online surveys or experiments drew on source data from MTurk or a similar platform.

Although the use of crowdsourcing platforms has been embraced by many academics, concern has also been voiced that they constitute “digital sweatshops” (Pittman & Sheehan, 2016, p. 260), and are a “poorly paid hell” (Semuels, 2018) in which participants are vulnerable to exploitation. Nonetheless, in a large-scale survey conducted of Turkers—that is, individuals who work on MTurk—38% felt “extremely positive” about the platform. Indeed, some declared in open-ended comments that they “love[d] working on MTurk,” and considered it a “Godsend” (Mehrotra, 2020). Clearly, crowdsourced research platforms *can* offer benefits to both researchers and participants. However, increased use and reliance on such platforms compels us to question how crowdsourcing platforms can be used effectively and ethically.

In what follows, we examine this question through a critical interpretive lens. We begin by discussing reasons for which researchers might be both wary of and enthusiastic about crowdsourced research, before highlighting factors that researchers who use crowdsourcing platforms should consider. Using principles of qualitative inquiry to preserve and amplify the voices of experienced Turkers, we then analyze a large corpus of open-ended responses in which Turkers describe how requesters can design online studies in such a way as to minimize the potential for frustration. In so doing, we hope that this will also enhance the degree to which researchers are able to operate in ways that recognize the essential humanity, agency, and contributions of crowdsource workers in general, and Turkers in particular (Gleibs, 2017). Ultimately, by considering Turkers’ descriptions of their frustrations in light of findings from the (web)-survey methods literature, we arrive at a series of suggestions for practice that may both improve the lot of crowdsource workers and improve the quality of data obtained by researchers.

## Reasons to be cautious about crowdsourced research

Researchers may be concerned that participants recruited via platforms such as MTurk may not be entirely “on the level.” Findings from some studies can appear to justify such concerns. For instance, by asking participants to report numbers obtained via rolls of virtual dice (which were linked to financial incentives) and comparing these responses to theoretically expected distributions, Suri et al. (2011) found evidence that Turkers misreported the results of their rolls even when the monetary incentive for doing so was small.

There is also evidence that a nontrivial proportion of Turkers misrepresent demographic and personality characteristics to gain access to studies from which they would otherwise be disqualified (e.g., Chandler & Paolacci, 2017; Siegel & Navarro, 2019), which means that researchers may legitimately question whether Turkers can be counted on to be who they say they are. In one multi-study report, for example, between 24% and 83% of respondents in a given study were identified as probable imposters (Wessling et al., 2017). The risk of researchers mistakenly believing they are actually studying a narrowly defined population of interest is thought to be amplified when recruiting from “a population that has limited representation on MTurk” (Siegel & Navarro, 2019, p. 246). In a recent study, for instance, Burnette et al. (2022) sought to recruit a sample of over 2000 transgender persons via MTurk to validate and establish norms for widely used measures of eating disorder symptoms. However, despite incorporating several tactics to increase the chances of gathering valid data, the research team was forced to abandon the planned project. Of 2413 respondents who consented to participate, passed attention checks, and did not complete the study implausibly quickly, 1060 provided inconsistent data with respect to their gender identity. Burnette et al. believed this cast doubt on whether they were, in fact, transgender, which was the primary criterion for inclusion in the study.

Despite the challenges that can beset collecting data via MTurk (and other crowdsourcing platforms) there *are* steps researchers can take to reduce the possibility that their sample comprises and is compromised by imposters (Wessling et al., 2017,1). Similarly, although some researchers have experienced challenges to data integrity as a result of bots (i.e., “malicious software” that auto-generates “indiscriminate responses to survey questions”; Roman et al., 2022, p. 1) and the use of server farms, strategies exist to detect fraudulent responses (Chmielewski & Kucker, 2019).

A different sort of challenge to researchers has become more pressing as the use of crowdsourcing becomes widespread. In the early days of crowdsourced research, there was only “a handful of respondents who participate habitually” (Berinsky et al., 2012, p. 366). Now, however, participant non-naïveté can be problematic. One of the “selling points” of the MTurk platform is the sheer number of potential participants to whom researchers have access. Amazon’s own data tracking suggests that there are as many as 750,000 unique monthly visitors to MTurk (Hitlin, 2016). Nonetheless, the number of participants to whom any given research group has access is likely to be orders of magnitude smaller, with Stewart et al.’s (2015) analysis suggesting that “a typical laboratory can access about 7300 workers” (p. 479), many of whom have already completed thousands of tasks on MTurk (Chandler et al., 2014; Harms & DeSimone, 2015). Such findings raise concern that as Turkers complete more and more studies, they become familiar with widely used stimuli and measures, thereby distorting research findings and—perhaps—increasing their potential to find Turking frustrating as they become more experienced survey-takers.

Another reason researchers may have reservations about carrying out crowdsourced research is that they may question whether Turkers—as a population—are attentive and diligent participants. Lending credence to such concerns, some researchers *have* found Turkers to be less attentive to experimental materials than participants recruited in more traditional ways (Goodman et al., 2012), to provide such rapid responses as to raise questions regarding the trustworthiness of the data (Harms & DeSimone, 2015), or to multitask while completing human intelligence tasks (i.e., “HITs”; Chandler et al., 2014). Several studies provide useful counterpoints, however. For instance, Necka et al. (2016) found that although Turkers may engage in undesirable respondent behaviors, they do not do so more frequently than participants recruited by other means. Furthermore, Hauser and Schwarz (2016) conducted a series of three studies from which they determined Turkers to be “*more* attentive to instructions than…college students” (p. 400; emphasis added).

## Reasons to be enthusiastic about crowdsourced research

Early meta-research sought to compare the demographic composition of MTurk samples to that of other convenience samples and to “gold standard” representative samples. Findings suggested that while MTurk samples did “not perfectly match the demographic and attitudinal characteristics of the US population,” neither did they “present a wildly distorted view” (Berinsky et al., 2012, p. 361). Moreover, several studies indicated that MTurk samples were considerably more demographically diverse than the typical American college sample that is often recruited for social scientific research (e.g., Berinsky et al., 2012; Buhrmester et al., 2011; Casler et al., 2013).

Researchers have also determined that the quality of data obtained via MTurk compares well to that gathered via professional marketing research companies or from college students with respect to measures of engagement, indices of test-retest reliability and internal consistency, and measures of criterion validity (Kees et al., 2017; Peer et al., 2014; Shapiro et al., 2013). Moreover, experimental effects and decision-making biases that have been well documented in laboratory or campus settings have been replicated with crowdsourced samples (Berinsky et al., 2012; Goodman et al., 2012; Paolacci et al., 2010; Peer et al., 2017), and researchers who have compared MTurk and college student samples have deemed the results “almost indistinguishable” (Casler et al., 2013, p. 2156). Attesting to the high degree of correspondence between results obtained via MTurk and those obtained in other ways, Stewart et al. (2017) observe that “in the many-labs project, the pattern of effects and null effects for 13 social psychology and decision-making coefficients corresponded perfectly between concurrently collected student and MTurk samples” (p. 741).

Finally, researchers’ confidence in the quality of data that can be collected via MTurk may be bolstered by the fact that the platform features a built-in system of only allowing workers with a sufficiently high reputation to participate in studies. Particularly in the relatively early days of MTurk research, findings indicated that academics could safely rely on participants’ MTurk reputation score as a means of assuring data quality (Peer et al., 2014). However, it is important to stress that this reputation score is only useful to the extent that requesters actually take the time to provide a meaningful rating of the work provided by workers. As Ahler et al. (2021) point out, although it may be relatively easy to provide a positive or negative rating for a participant who performs a task that can be completed in an objectively “right” or “wrong” way, it is more difficult for researchers to rate a participant who provides an opinion or responds to measures that are designed to capture subjective perceptions. Moreover, a researcher who only uses MTurk quite infrequently “has few incentives to sink resources into monitoring quality; instead her investment is typically capped at the payout rate” (p. 3). As such, Ahler et al. caution that a worker’s HIT approval rate is very likely to be an upwardly biased indicator of reputation.

In sum, we believe that, in general, research speaks favorably of the quality of data that can be obtained from respondents on the MTurk platform. Admittedly, samples drawn from MTurk cannot be treated as representative, but they are particularly well-suited for “conducting internally valid experiments” (Berinsky et al., 2012, p. 361).

## The experience of Turking

Researchers have asked and answered numerous questions regarding how best to make use of MTurk and other crowdsourcing platforms. Unfortunately, the perspective of those constituting the crowd has largely been missing, although some researchers have published auto-ethnographic reports of their own experiences as temporary Turkers (Schmidt, 2015), or considered the ethical treatment of crowdsourced participants (e.g., Chandler & Shapiro, 2016; Gleibs, 2017; Paolacci et al., 2010).

One area of concern regarding the ethical treatment of Turkers (and members of similar platforms) is compensation. Brown (2015, para 7) observed that “researchers are going to have to face up to the fact that by using MTurk, they are typically exploiting sub-minimum wage labour.” His remark was not hyperbolic, for an analysis of 3.8 million HITS showed that just 4% of a sample of 2676 Turkers earned more than $7.25 per hour (Hara et al., 2017). This is particularly concerning because many individuals participate in crowdsourced work because there is no other work available to them (Marder & Fritz, 2015*;* Mehrotra, 2020*;* Semuels, 2018), and a quarter of Turkers canvassed by Pew Research reported that they derived “all” or “most” of their income from MTurk (Hitlin, 2016). Highlighting the sensitive issues surrounding compensation, Burnette et al. (2022) report receiving emails from Turkers that speak to the pressure they felt to be approved for payment, and describe one email in particular in which a worker “pleaded for their HIT to be approved because they needed the money to feed their child” (p. 265).

There have been calls to ensure Turkers receive at least the local minimum wage (e.g., Harms & DeSimone, 2015), and perhaps follow the lead of a rival platform, *Prolific,* that will not allow researchers to pay below a certain level, sanctions requesters who underpay relative to the time a task takes, and—if requests to retroactively increase payment to acceptable levels go unheeded—suspends researcher accounts. Gleibs (2017) went a step further, suggesting that journals “could require authors to pay minimum-wage scale (from the respective participants’ country of residence) incomes to crowdsourced participants” (p. 1338) and recommended that ethics boards should weigh “standards of fair pay and employment protection” when determining whether a study meets standards for ethical conduct.

Researchers must also consider several other issues related to compensation. For instance, although it is normative to compensate participants who withdraw from a study (either in whole, or proportional to the time they have invested in a study), crowdsourcing platforms often make it difficult to pay participants who do not complete a project (Burnette et al., 2022; Gleibs, 2017; Paolacci et al., 2010). Indeed, non-payment for work completed in good faith has been identified as a perennial problem facing Turkers, given that the platform allows requesters to reject work and refuse payment while still retaining use of the data obtained (Chandler & Shapiro, 2016; Paolacci et al., 2010).

Other ethical principles also need to be considered. Unless researchers accurately report how much time participants will need to spend on a HIT, and the nature of the work involved, it is difficult to claim that respondents are able to provide informed consent (Paolacci et al., 2010). Researchers must also recognize that the nature of online research makes it difficult to know whether their studies have caused harm to participants (Chandler & Shapiro, 2016). Mehrotra (2020, paras. 32–33), for example, heard from workers who had felt “emotionally traumatized by an academic survey,” who experienced “intensely negative feelings,” and who were “brought to tears” by being asked to recall painful experiences. However, unless these workers contacted researchers to relay these experiences, the researchers would have no way of knowing that participating in their studies had caused such distress.

Despite the legitimate concerns that have been expressed over the working conditions experienced by Turkers, a recent large-scale study offers an encouraging counterbalance. From a survey of over 4000 Turkers in the United States, Moss et al. (2020) found that they were not more financially vulnerable than were members of the wider US population. Moreover, they generally did not find the experience of Turking to be stressful, nor did they often report finding their working conditions (or requesters) to be abusive. In fact, they found the benefits offered by Turking sufficiently appealing that they “would not trade the flexibility of MTurk for less than $25 per hour.”

It should also be emphasized that Turkers themselves seem to take their work seriously. Marder and Fritz (2015, para 74) spoke with 100 “Super Turkers,” and learned that many “reported a degree of pride in their work, despite the tedium and lousy pay. And notably, despite the lack of oversight, they weren’t even tempted to game the system.” Marder and Fritz go on to point out that although some researchers might worry that their study could be compromised if participants share information about it, the official rules for a popular online forum for Turkers (“Turker Nation”) specify that there must be “no disclosure or discussion of attention memory checks. No discussion of survey content, period. That can affect the results” (Marder & Fritz, 2015, para 76).

### Research purpose

Our own research has increasingly drawn on crowdsourced samples. The impetus for this study comes from our desire to use the crowdsourcing platforms such as MTurk in ways that recognize and respect the contributions (and indeed, basic humanity) of the people who complete our studies. We therefore sought to gain direct insight from Turkers about their frustrations with—and recommendations for—crowdsourced research participation. The following research question guided our inquiry.

- RQ: What can we learn from the frustrations Turkers report about their participation in crowdsourced research?

## Method

The first author was preparing to collect data for a study with specific eligibility criteria. To minimize the risk of character misrepresentation, he followed Wessling et al.’s (2017) “two-survey process” by posting a paid pre-screening HIT to MTurk (via CloudResearch) with a view to inviting eligible persons to be involved in the substantive part of the study. As part of this pre-screening HIT, the researcher included an open-ended question inquiring about participants’ recommendations for reducing frustrations for Turkers. Their responses provided rich insight into the experiences and frustrations of Turking and served as data for the current study.

We framed our study within a critical interpretivist paradigm. By applying a critical paradigm, we were able to focus our attention on issues of power, values, exploitation, equity, and fairness (Lindlof & Taylor, 2011) experienced by Turkers. By also applying an interpretivist frame, we were able to retain a sharp focus on the social realities, knowledge, and lived experiences of the participants (Lindlof & Taylor, 2011). This approach allowed us to preserve and amplify the voices and experiences of Turkers, to provide empirical evidence of their frustrations with this type of work, and to use their responses to guide how we design our own research studies so that they demonstrate both scientific integrity and respect for respondents.

### Participants

The pre-screen was accessed 1509 times. Removing cases where nothing was entered for one or more of the open-ended questions and eliminating nonsense or bot-like responses to the open-ended questions reduced the number of useable responses to 1369. Of these, 50.6% (*n* = 692) of respondents were female, 49% (*n* = 671) were male, and .4% (*n* = 5) identified as being outside the gender binary. Participants’ mean age was 37.73 years (*SD* = 11.69; range = 18–82).

Participants were asked to identify which ethnic group(s) they belonged to; given that they were able to check multiple ethnicities, percentages sum to more than 100%. The majority of respondents (1093) identified as White (79.8%). An additional 120 (8.8%) respondents identified as Black or African American; 123 (9.0%) as Asian or Asian American; 83 (6.1%) as Latino/Hispanic; 20 (1.5%) as American Indian or Alaskan Native; 9 (0.7%) as Native Hawaiian or Pacific Islander; and 5 (0.4%) as Middle Eastern. Nine people (0.7%) marked the “other” box.

### Procedures

As Wessling et al. (2017) point out, “it is important that the screening question[s] be masked by other questions” (p. 221). So that participants could not easily intuit that this was, in fact, a pre-screener for a subsequent study, the pre-screener questionnaire was titled “Improving my surveys for Turkers.” Participants were asked how many academic surveys they thought they had completed on MTurk during the previous week (none; 1–5; 6–10; 11–14; 15–20; 21–25; 26–30^2^; and over 30). The modal response was “over 30” (*n* = 641; 46.9%). We asked participants who checked the “over 30” response to estimate the number of surveys they had completed during the preceding week. The mean number was 105.88 (*SD* = 162; median = 69.50). Excluding the estimates of 16 respondents whose responses exceeded 486 (i.e., 3 *SD* above the mean) reduced the mean to 85.93 (*SD* = 60.14; median = 65.00)^3^.

Participants were also asked two open-ended questions regarding their experiences of Turking. Although there was no character limit imposed on the length of responses, respondents were told that they only needed to write a sentence for each^4^. Participants were paid 50 cents, for what was projected to be a three-minute study^5^. In this manuscript, we focus our analysis on participants’ written responses to the question “*How could academic surveys on MTurk be designed so that frustration for Turkers is reduced/minimized*?”

#### Coding and data analysis

We used an iterative coding procedure. To begin, each investigator read the corpus of data to get a broad, holistic sense of participants’ experiences. Then, two members of the research team conducted the primary, inductive coding and analysis, reserving the principal investigator (PI) for the coding confirmation check. In the first phase of coding, we reviewed each of the 1369 responses to determine what would be the unit of analysis. Because single responses could contain several frustrations and recommendations, we established the unit of analysis to be each discrete frustration or recommendation mentioned within each response rather than the entire response. Specifically, each item (concept) in a string of frustrations or recommendations was coded individually.

The first cycle of analysis began with open coding. Open coding is a process of assigning a unique label to each chunk of data that addresses the research purpose. Rather than reducing the data through assignment to predetermined categories or codes, open coding is an inductive approach that expands the corpus of data to allow analysts to identify nuanced findings and examine the unique parts that make up the whole. An advanced graduate student trained in qualitative analysis open coded the initial 1369 responses, applying in vivo codes as often as possible. In vivo codes use the participant’s own words or phrases as the name of the code. This allows the analysts to foreground the participant’s meaning during coding while mitigating the compulsion to impose their interpretation too early. The first cycle of open coding concluded after every participant response was assigned at least one code. This process yielded 463 unique open codes with 2310 references (units of data assigned to codes).

We then engaged in iterative rounds of second-cycle coding. Second-cycle coding is a process of organizing similar open codes, and later, clusters of open codes, into larger, increasingly abstract thematic categories—each time assigning a descriptive label reflective of the content to the emergent categories. During second-cycle coding, we first clustered open codes into 31 categories and then further collapsed them into 22 categories that we then organized into six overarching themes (see Table 1). At the end of the second cycle, we engaged the PI to confirm the coding reliability. The PI was given a table with a conceptual definition and prototypical exemplar for each category and asked to code 10% of the total units of analysis (*n* = 232) into those categories. As an established and conservative approach to evaluating intercoder coding reliability, we used Krippendorff’s *α* (Krippendorff, 2012). Krippendorff (2012) suggests that *α* above .667 is tentative and *α* at or above .8 is preferred. Intercoder reliability was excellent in our study, as evidenced by α = .916 for the 22 subcategories, and α = .955 for the overarching themes.

**Table 1:** (Sub)themes of Turking frustrations

(Sub)theme	Number of references	Percentage
Difficulties with survey design and accessibility		**37.84%**
Structural and visual issues	384	16.62%
Did not have a progress or completion bar	117	5.06%
Should be shorter	108	4.68%
Should allow more time to complete	85	3.68%
Should be proofread	55	2.38%
Survey accessibility	74	3.20%
Should make it more interesting and engaging	51	2.21%
Frustrations with question design		**24.16%**
Repetition of questions	218	9.44%
Question quality	132	5.71%
Providing written responses	111	4.81%
Store answers to common questions in profile	57	2.47%
Some questions should not be asked	40	1.73%
Fair pay for fair work		**13.29%**
Did not pay well	277	11.99%
Did not pay for qualification questions or failed attention checks	25	1.08%
Should indicate how long payment will take and pay quicker	5	0.22%
Frustrations due to qualification checks, attention checks, and confirmation codes		**11.65%**
Troubles with or about confirmation codes	111	4.81%
Troubles with or about qualification checks	83	3.59%
Annoying attention checks	60	2.60%
Should be more careful when rejecting work	15	0.65%
Desire for clear, accurate, and convenient communication between workers and researchers		**10.78%**
Clarity and accuracy of the HIT	232	10.04%
Should enable more convenient communication between requestors and workers	17	0.74%
No frustrations		**2.29%**
No frustrations	53	2.29%

The bolded entries reflect the percentages for the overarching category

## Findings

For MTurk workers, frustration is commonplace. Indeed, there were only 53 instances (2.29%) of participants listing *no frustrations*. Many participants wrote about multiple frustrations, with most listing at least 2 (*M* = 1.7).

### Design features and platform limitations cause Turker disengagement

Our first theme, *difficulties with survey design and accessibility,* represents the most frequently mentioned source of frustration—design issues (*n =* 874; 37.84% of responses). The chief source of frustrations in this category concerned the structural and visual/aesthetic design of surveys (*n* = 384; 16.62% of all coded responses).

Frustration with the structural and visual/aesthetic design of surveys was manifested in various ways. Participants frequently reported feeling that they had been consigned to “bubble hell.” That is, they felt “daunted” or put off being presented with a page (or pages) of endless “bubbles” (i.e., radio buttons presented in grid form) to click. Participants also wanted both “bigger” and “fewer” bubbles on a page, and often complained about surveys not having go-back buttons. In short, they lamented the apparent prioritization of function over design, with one respondent explaining: “I like to take surveys that appear to be streamlined and well designed. Sometimes the appearance is cluttered. I’ve returned hits that have too much information or tasks on a single page. It makes me lose focus.” Importantly, however, respondents noted that the visual presentation of material also impeded functionality. For instance, Turkers found it frustrating when requesters failed to use “constant labels” for scales or did not use a “consistent question (or answer) format.” Participants also emphasized the importance of making sure the wording for a response (e.g., “highly satisfied”) is always visible in addition to the number (“5”). One specific recommendation was that researchers “highlight choice bubbles with their appropriate columns so that the workers do not have to constantly scroll back to the top of the page to see what each column and bubble represents.”

Another important frustration raised by participants concerned the length of studies, which participants often believed were too long and could (or should) be shorter (*n* = 108; 4.68%). This issue was compounded for many participants both by the lack of a progress bar to indicate how much of the survey remained (*n* = 117; 5.06%), and the fact that the time allotted to complete the task prior to being “timed out” was insufficient (*n* = 85; 3.68%).

Broad issues of survey design were also identified by participants who noted their frustration with typographic errors resulting from a lack of proofreading (*n* = 55; 2.38%). “I think that proofreading/beta-testing surveys before they are released would help minimize frustrations,” wrote one participant. Another participant commented, rather pointedly, that “There’s little more galling than running into attention checks in a study that the researchers appear to have barely paid attention to themselves.” It is notable, we think, that although researchers may question the attentiveness and carefulness of participants, numerous participants eloquently articulated similar frustrations regarding requesters.

Participants also recommended researchers design surveys with a view to making tasks more interesting, engaging, and even entertaining (*n* = 51; 2.21%). This was not simply a matter of aesthetic preference: Turkers felt that this would improve their overall experience during a survey, thereby facilitating their attentive completion of tasks. In the words of one respondent:

A bit of humor helps. I recently took one where some ridiculous questions/answers were mixed in, and it brightened my mood. Also try to mix things up so that it’s not just page after page of questions using the same scale. Video and music is always great to see since it's more interesting than just reading a lot of text.

Finally, a relatively small proportion of frustrations related to issues of survey accessibility deriving from platform limitations of MTurk (*n* = 74; 3.20%). These largely related to issues of compatibility with and/or requirements for particular devices, software, or browsers (e.g., “tell me it requires Firefox ahead of time so I’m not returning it” [i.e., the HIT]).

### Frustrations with questions demotivate Turkers

The theme, *frustrations with question design,* captures the second most frequently mentioned source of frustrations (*n* = 558; 24.16% of all coded responses). Participants expressed frustration with the perceived repetitiveness of questions, unhelpful response options and formats, and overall question quality or content.

Repetition of questions within and across surveys topped this list of frustrations (*n* = 218; 9.44% of all coded responses). Although some participants recognized that researchers may need to measure complex constructs in multiple ways or with multiple items, “asking the same questions in tons of different ways” was a persistent complaint. One respondent observed, for example, that “Spending a huge amount of time answering the same questions, only worded slightly different, is a real headache,” noting that while they “underst[ood] the reason behind it,” they found that “when I have to answer the same general question 10 times, my attention level drops drastically.” Likewise, another Turker explained that “Too many questions are…pretty much the same…but differing in tiny ways that are not relevant. Or maybe they are, I don’t know, but they tend to get boring.” Notably, some participants not only felt wearied by repetition, but took personal offence at it. One individual wanted researchers:

To not repeat questions in different ways, but instead phrase questions meaningfully and trust that people will respond honestly. I feel a bit demotivated when I see the same question asked more than once, like the integrity of my data is in question.

The quality of questions in general (and quality of response options in particular) were another source of frustration for Turkers (*n* = 132; 5.71%). Some complained about options that were inexhaustive (“should provide more options of answers”) or that did not include a “not applicable” option.

A number of participants reported that they found it frustrating to be asked to complete open-ended responses or longer writing tasks (*n* = 111; 4.81%). Several respondents simply said that they did not like any writing tasks, commenting that they should be avoided or used sparingly. In one Turker’s words, “Requesters have to realize…that Turkers loath [*sic*] any survey that has an extended amount of writing in it. So either design your surveys so you aren’t asking open-ended questions, or incentivize Turkers to answer them by increasing pay.”

A fairly common frustration expressed by participants concerned the need to complete “boilerplate questions.” More specifically, participants found it annoying to have to provide basic demographic measures or complete common scales every time they accepted a HIT, with some noting they would prefer “answers to some questions…[to be]…stored in the profile and only be taken once” so that “repeated demographic or personality questions [could be] auto filled.” There were 57 references to this issue (2.47% of all responses). Finally, participants felt that “some questions should not be asked” (*n* = 40; 1.73%). They disapproved of having to supply “personal” or “unnecessary private information” about age, race, finances, and so forth, in some cases perceiving this information to be outside of the scope of their task.

### Turkers desire fair pay for fair work

The third theme, *fair pay for fair work* (*n =* 307; 13.29% of all coded responses), relates to the amount, fairness, and timeliness of payment. This category was dominated by the simple frustration that Turking did not pay well (*n =* 277; 11.99% of coded responses). As one Turker wrote, “Better pay is always needed. A fair wage would compensate for any type of task.” Another noted that although, for them, poor compensation levels merely affected their “pocket money,” others were affected in more profound ways. In their words, “$0.10/m is often recommended as good pay but $6/h is an awful pay rate. I turk part time for pocket money but that rate is below minimum wage and some people do rely on that money completely.” Interestingly, some respondents observed that poor pay affected *researchers* negatively by forcing crowdsource workers to accept multiple HITs in order to cobble together a reasonable hourly wage, which necessitated rushing to complete tasks. Explicitly noting the association between pay and data quality, one Turker stated:

The pay is what makes me interested in surveys. Upping the pay is almost always appreciated—and will almost always ensure better, careful results. People on Mturk tend to try to make hits pay out to a decent hourly. If the hit is underpaid, they will probably rush through…whereas if it’s a generous payment they’re going to want to give the requester good data. It’s just how things are here.

It was important to participants that compensation be commensurate with the nature and quantity of work required by a HIT. For example, they suggested that longer surveys and surveys with writing prompts or other engagement activities should be better compensated. Reflecting this, one person stated that “I enjoy academic surveys as I assume that my opinion truly matters. It’s only the long ones with very little compensation where I feel…taken advantage of.”

A number of participants highlighted the injustice of not being compensated for completing (often lengthy) qualification and screening questions only to then be deemed ineligible for a study. Similarly, participants who devoted significant time to completing a survey, but who failed to appropriately respond to an attention check question or activity, were frustrated by the lack of payment for their engagement. From their perspective, they spent time on the task and should be compensated for the work they did complete. Twenty-five of the coded responses (1.08%) reflected frustration concerning not being paid for qualification questions or being denied payment on the basis of failing attention checks. A smaller proportion of responses in this theme (*n* = 5; 0.22%) also voiced frustrations related to a lack of timely payment. Turkers were leery about accepting HITs that did not indicate how long payment would take and reported feeling frustrated when payment took longer than seven days: There was a strong desire for uncomplicated, transparent, and “quick” compensation.

### Confirmation codes, attention checks, and (dis)qualification processes disrupt and distress Turkers

The fourth theme, *frustrations due to qualification checks, attention checks, and confirmation codes,* represents 11.65% of all coded responses (*n =* 269). Having to spend time searching for confirmation codes was the most oft-occurring complaint in this theme (*n* = 111; 4.81% of all coded responses). Simply put, participants wanted requesters to display confirmation codes clearly and prominently, and to ensure that they are actually provided.

Turkers also found it frustrating when there were “too many” qualification questions, or when such questions appeared late in a task (*n* = 83; 3.59%). As one participant explained, “If there are qualifications, list them ahead of time. I sometimes take surveys then am rejected afterwards because I didn’t meet screening criteria when there were none listed.” Annoyance with attention checks^6^ featured almost as prominently (*n =* 60; 2.60%) and elicited more detailed feedback. Turkers felt that attention checks disrupted the research process and distracted workers from their task, as seen in the examples below:

The frequency of attention checks could be reduced, especially when they are placed in a long psychological survey where it breaks up the flow of reading the questions.

Don’t over complicate attention checks, whenever I see a complicated attention check all I think about is the next one coming. It takes the focus off of the survey and adds unnecessary anxiety.

It is important to note that some participants found attention checks to be not only distracting or anxiety-provoking, but *patronizing*. One participant, for example, wrote that “attention checks could be replaced with smarter and less condescending strategies to ensure data quality (e.g., consistency check, 2-step recruitment).” At the very least, respondents noted that “new and exciting attention checks could help.”

Finally, frustration was expressed over the process of disqualification (*n* = 15; 0.65%). Participants pointed out that having their work rejected may lower their reputation rating. They implored requestors to be careful when rejecting work, and to provide reasonable explanations for doing so. As one Turker put it:

Please don’t reject the work unless absolutely necessary. We want to do a good job and if a requester rejects for no reason and won't talk to us, we worry about keeping up our good percentages. It’s best to reject the survey at the beginning if an attention check is missed. Just stop us from getting bad marks instead of letting us do the whole survey then rejecting it.

### Turkers want clear, accurate, and convenient communication with requestors

The fifth theme, *desire for clear, accurate, and convenient communication between workers and researchers,* centers on frustrations related to communication between Turkers and requestors (*n =* 249; 10.78% of all coded frustrations). Failure to communicate accurate time estimates was the most prominent category within this theme, although the broader point that emerged (*n =* 232; 10.04% of all coded frustrations) was simply that Turkers wanted clear, accurate, and convenient communication with researchers from the point at which they were recruited to the point at which they were paid.

Turkers rely on the information provided about a HIT to make a decision about whether to accept it. As such, they wanted an “accurate and honest” communication up front about how long a HIT will take, what they are expected to do (e.g., providing written responses or accessing external URLs), and whether there were technical requirements for completing a HIT, such as using a certain browser or downloading additional software. For some Turkers, poor or inaccurate communication was stressful as well as frustrating.

The biggest annoyances are misleading time estimates and surveys not mentioning there is writing involved in the description. I think requesters should have a few people take their survey first to get an accurate time estimate and then base the pay on that estimate, because when they say “This survey takes 10–15 minutes” and it winds up taking only 2 minutes or so, I get paranoid thinking they might reject me for working too quickly.

A relatively small number of the frustrations expressed by Turkers (*n =* 17; 0.74%) concerned problems relating to the lack of convenient ways of communicating with requesters. For example, Turkers reported difficulty communicating with researchers especially when they “have a problem,” with one Turker recommending that it needed to be “easier for requesters and workers to communicate about hits.” Others expressed frustration with the lack of accessible contact information and reported that researchers were unresponsive to their inquiries. For example, one Turker wanted “a better way to report if a code was not found; half the time [I] never heard back from requesters.”

## Discussion and recommendations

We begin our discussion with a summative comment from one participant that encompasses many of the frustrations represented in the thematic findings.

Oh, thank you for asking! First, make certain you’ve allowed enough time. This is one top peeve for turkers. Second, just as important—don’t lie or carelessly throw out a time estimate for your study, that winds up being incorrect. Requesters will be crucified in their reviews. Also, make sure the directions are crystal clear. Ambiguity is incredibly annoying! Also, very important, do not forget the code! Also, make sure every part of your hit works before you let it loose on the community. We also hate it when questions are super repetitive or when there is what we call a “bubble hell”. Bubble hell does not promote interest in your study. It promotes boredom and a desire to leave the study. I think that’s all I can think of.

Even before the outbreak of COVID-19, crowdsourcing platforms had become the primary means of data collection for some researchers. They also provided a supplementary (sometimes primary) source of income—as well as opportunities to contribute to scientific endeavors—for participants. Now that once-abstract terms such as “lockdown” and “social distancing” have come to define many individuals’ experiences during 2020 and 2021, it is more important than ever to reconsider the symbiotic relationship that exists between researchers and participants as they navigate vastly changed social, academic, and economic environments. By examining and extrapolating from the frustrations voiced by MTurk workers, we identify several factors researchers could (and, we believe, *should*) take into account when designing studies to be conducted via crowdsourcing platforms. Having had the opportunity to read almost 1400 Turkers’ comments on what they find frustrating about their work—and for many, it *is* work, which has attendant implications for the degree to which researchers should strive to be good employers—we urge researchers to reflect on how their online research practices can better establish a climate of trust and demonstrate respect for participants’ time, voice, and labor.

Before turning to the specific recommendations, we wish to highlight a key issue that underlies many of the frustrations expressed by Turkers: Regardless of how individual researchers perceive themselves as treating participants from MTurk and similar platforms, participants’ discourse frequently revealed their perception that researchers (as a collective) do not value their time, intelligence, knowledge, or capabilities. To some degree, unfortunately, these assumptions may be justified. Consider, for example, that an early study (Buhrmester et al., 2011) explored whether or not participants could be recruited to complete a 30-minute survey when offered just two cents to do so. In fairness, this was only one of several aims, and these authors’ analyses indicated that—in the early days of MTurk—participants were intrinsically motivated (e.g., for enjoyment) rather than extrinsically motivated. Nonetheless, even if Buhrmester et al.’s *intent* was to highlight that “workers are not driven primarily by financial incentives” (p. 4), we suspect that in many of the eleven-thousand-and-counting manuscripts that have cited this article, this was not the obvious message. Rather than interpreting Buhrmester et al.’s findings as suggesting that researchers might consider how the design of their studies can capitalize on participants’ high levels of intrinsic motivation, we believe that what many have taken from this report is that “workers are willing to complete simple tasks for virtually no compensation” (p. 4).

### Recommendation 1. Recognize that Turkers are sensitive to the substance of a HIT

About a fifth of frustrations concerned what Turkers were asked to do during HITs. Turkers were frustrated by questions that required written responses (4.81%) (or were poorly formulated; 5.71%) and irked by attention checks (2.6%). They felt requesters asked inappropriate questions (1.73%) (or ones whose answers should be available from a centralized repository; 2.47%) and found HITs to be uninteresting (2.21%).

#### Open-ended questions

We recommend researchers use open-ended questions sparingly and signal their use in HIT descriptions. This is especially important for Turkers using mobile devices and for Turkers who accept a HIT not expecting to have to write anything and only find doing so is required after investing time in the HIT. As one participant put it: “Announcing that there will be an open-ended question is nice, but not always needed. However, going through 5–15 minutes of a survey then seeing a couple of essay questions with a large word count or several writing prompts isn't fair. It feels like my information is being stolen as I will return many of these hits.” Other Turkers did not find open-ended questions inherently frustrating but resented how much time and effort they required given the rate of pay. Commenting on HITs where requesters ask for an essay “as vivid and detailed as possible” but only offer a “30 cent payoff,” one Turker noted that “I used to do these in the early days before I knew better. Now I just return them.”

Open-ended questions can put a high cognitive load on participants (Zuell et al., 2015) and adversely affect completion rates (Liu & Wronski, 2018). Indeed, Crawford et al. (2001) found that almost a third of respondents who quit a survey did so when shown the first of a set of open-ended questions. The risk of nonresponse can, however, be partly mitigated by clarifying why an open-ended question is being asked, stressing the value of a participant’s response, and avoiding the use of dauntingly large text boxes (Müller et al., 2014; Zuell et al., 2015).

#### Attention checks

Only 2.6% of responses directly concerned attention checks (ACs). However, the language used to describe them was strong (e.g., *bullshit, condescending, deceptive, disrespectful, gotcha, malicious, sneaky, trick, unfair*), which suggests that using ACs risks creating what one Turker described as an “adversarial” relationship between researchers and respondents. Downs et al. (2010) argued that many ACs violate Gricean norms of communication, “requiring careful attention to normally predictable information…set[ting] a tone of distrust for the remainder of the task” (p. 2400). ACs certainly generated “implicatures” among some of our respondents, who found them demeaning and demoralizing, and attributed their use either to researchers’ laziness or lack of consideration.

Our participants did not call for researchers to stop using ACs entirely. Indeed, some noted that they understand their necessity (“I understand and appreciate the need for attention checks. I agree with attention checks”; “You need to maintain the quality of your data, so attention checks…are necessary”). They did, however, voice multiple, specific frustrations with ACs. For instance, it annoyed Turkers when researchers included too many ACs or let them continue a study after failing an AC only to later reject their work. Turkers were also frustrated when researchers put ACs in consent forms or demographic pages that “people have seen 1000 times,” don’t ensure ACs work properly (“select option X…no option X to select”), test recall of distal information rather than attentiveness, or “fake-out” participants. As one Turker explained:

There will be a passage of text, and in the middle it will say, actually disregard that, please type *xyz* in the answer box…Most people would stop reading here and type *xyz* because they believe they’ve found an attention check…However I’ve seen some requesters add contradicting attention checks later on the same page, i.e. ‘disregard the passage and the former instructions and actually, ~actually~ for real this time type *abc*’.

Academics often infer data quality or study engagement from participants’ responses to ACs. However, including ACs may introduce new threats to validity (Hauser & Schwarz, 2015) and weed out only the “most egregious” of participants (Downs et al., 2010, p. 2400). We believe that—by implying disdain and distrust—researchers’ unthinking use of ACs can induce ill will among participants. We recommend researchers consider carefully whether and how to use ACs instead of taking for granted that they will enhance data quality.

#### Overly familiar content

Turkers find it frustrating to complete the same survey items time and again. Therefore, it behooves researchers to balance their need to use validated measures with participants’ need for novelty. Doing so may alleviate participant frustration while perhaps also mitigating the threat to validity posed by participant non-naïveté (Chandler et al., 2014). Turkers also found it frustrating to have to provide the same demographic information in HIT after HIT, and some stated that they would rather complete a demographic profile that is automatically appended to submissions. Currently, Prolific lets researchers download certain demographic information about participants (e.g., sex, age), but this is not possible on MTurk. However, with researcher permission, *CloudResearch* adds questions to HITs to monitor the consistency in participants’ reported demographic characteristics over time. With appropriate privacy safeguards, Turkers may appreciate CloudResearch (or Amazon themselves) making “on-record” demographic information available to researchers so that they do not have to enter it so often.

#### Asking questions that shouldn’t be asked

Although MTurk prohibits requesters from asking for email addresses, phone numbers, social media handles, et cetera (https://www.mturk.com/acceptable-use-policy), some requesters do ask for personal data. And, perhaps because they fear losing access to work or have been induced (or coerced) into doing so by a high rate of pay or a bonus, some workers acquiesce to these requests. A respondent in Sannon and Cosley’s (2018) study, for instance, explained their reluctant decision to disclose personal information, saying they were “homeless at the time,” and, because they “really needed the money…[they] went and did it anyways” (p. 3).

Researchers who require participants to provide personal information are (on some crowdsourcing platforms) violating the terms of service. Just as importantly, they are violating participants’ right to privacy. And—if payment is perceived as being contingent on acquiescing to such information requests—they are likely violating ethical principles of non-coercion.

### Recommendation 2. Don’t underestimate the importance of stylistic elements of a HIT

A large proportion (19%) of frustrations related not to the substantive nature of a HIT but its presentation. Turkers often voiced discontent with seemingly banal structural and visual issues (16.62%) such as font size, having too few or too many questions per page, being confronted with “endless pages” of bubbles, as well as with poor proofreading (2.38%). Good aesthetic design should not be an afterthought: It can improve participants’ willingness to complete a study (Biffignandi & Bethlehem, 2021) and facilitate their processing of questions (Casey & Poropat, 2014). Poor aesthetic design, on the other hand can trigger “negative visceral responses and, thus, emotional reactions” from participants that reduces data quality (Mahon-Haft & Dillman, 2010, p. 43). One participant in our study noted, for example, that having “too many words on one page…makes me feel very overwhelmed.” Arguably, overwhelmed people may be relatively unlikely to provide high-quality data.

#### Grids and matrices

“Bubble hell” describes studies that feature lots of scale items in a grid or matrix, with responses being entered by selecting a radio button. Such formats may be especially onerous for participants using smartphones (especially if they must scroll for the whole matrix to be visible) (Biffignandi & Bethlehem, 2021; Dillman et al., 2014), and research suggests that lengthy bubble-based surveys may have higher rates of nonresponse and straight-lining (i.e., answering with a response set) (Liu & Cernat, 2018; Müller et al., 2014). Unfortunately, respondents in our study also reported frustration with alternatives to matrices (such as drop-down lists and sliders). This reflects findings that drop-down lists are more difficult and time-consuming for respondents to use than are radio buttons and can “result in more accidental selections” (Müller et al., 2014). It may not be possible (or desirable) for researchers to avoid using bubble questions. However, ensuring the meaning of response options stays visible and breaking up walls of text with white space may reduce participants’ sense that they have been consigned to bubble hell.

#### Pagination

Respondents rarely stated how many items per page they considered too many or too few, but even if they had, we doubt there were would have been agreement on an exact figure. Our view is that it is probably better to err on side of having too few items per page, because including high numbers of items on a single screen can increase nonresponse rates (Toepoel et al., 2009) whereas spreading items over multiple pages (and including clear section headers) can make it easier for participants to cognitively process survey questions (Müller et al., 2014).

### Recommendation 3. Reward participants’ time, help them manage it, and make good use of it

Turkers voiced frustration about several related issues: Compensation (hourly pay rate, 11.99%; unpaid screeners, 1.08%), obstacles to being paid (missing confirmation codes, 4.81%; getting rejected, 0.65%), excessive survey length (4.68%) and question repetition (9.44%), and difficulty in knowing whether a HIT is worth their time (inaccurate estimates of time required by HIT descriptions; absence of progress bars (5.06%). Collectively such frustrations constituted at least 37.71% of those raised^7^.

#### Remuneration, withheld pay, and rejected work

Crowdsourcing has changed the relationship between researcher and participant into one wherein “the requester is a client and the participant a contractor” (Gleibs, 2017, p. 1337). Turkers see completing HITs as a job and requesters as employers—albeit ones who do not always pay or treat them fairly. Whereas Turkers were once thought to be motivated mainly by nonfinancial factors (Buhrmester et al., 2011), “monetary compensation is now…the primary reason” for Turking (Litman et al., 2015, p. 519). This shift may explain why, despite early findings that the quality of data provided by Turkers was largely unaffected by rates of pay (Buhrmester et al., 2011), recent findings suggest that compensation *does* affect data quality (Robertson & Yoon, 2019). One very simple recommendation, therefore, is that researchers offer fair pay that meets community norms and appropriate local/regional level standards.

When Brawley and Pury (2016) asked Turkers to describe a time when they were dissatisfied with how a requester treated them, rejection of work was the dominant theme: As their reputation scores grant entrance to well-paying HITs, workers are protective of their scores and resent it when they are lowered as a result of unjustly being given “black marks” (i.e., rejections). In *our* study, fewer than 2% of respondents reported frustration with requestors not taking sufficient care when rejecting work or not paying them for work attempted. Still, we consider these to be important issues, especially in light of Brawley and Pury’s findings and the ethical implications of not paying for partial completions or rejecting participants’ work.

The ethics of rejecting work from Turkers and workers on other platforms is murky. In offline studies conducted in university classrooms and labs, participants are rarely rejected for poor performance or (presumed) inattentiveness (Gleibs, 2017). Further, invoking the right to withdraw without penalty is relatively straightforward in such studies: If a participant is uncomfortable, they can explain this to the researcher, leave the lab, and still expect to be compensated. Despite the wording often included in consent forms, this is less likely in the world of crowdsourced research. Complicating questions of whether and when it should be permissible for researchers to reject work^8^ is the fact that rejection is the only means of exercising quality control on MTurk. Researchers often allow only workers with a sufficiently high reputation score to participate in their studies. As such, they *rely* on their colleagues rejecting “poor” work so they, in turn, can be confident in the quality of the data they collect (Peer et al., 2014).

Two reasons for having work rejected irked our participants. The first was when work was rejected after failing an attention check, especially if they had already invested time in the HIT or were allowed to complete the HIT only to later be rejected. The second was when they were rejected for completing tasks too quickly. One participant wanted researchers to realize that “someone who does this all day every day is much faster at completing…and isn’t simply not paying attention.” Another noted that Turkers are “going to be faster than the graduate students you had pilot testing your survey” because “it’s their job,” and that they shouldn’t be “penalized” for this. Such sentiments echo Robertson and Yoon’s (2019) conclusion that “the amount of time…an MTurk participant spends on a task may be a poor proxy for effort” (p. 1656).

#### Progress bars

That more than 5% of frustrations concerned the failure to include a progress bar reflects that progress bars are appreciated and desired by respondents (Heerwegh & Loosveldt, 2006; Müller et al., 2014). To the extent that participants who may be on the verge of exiting a study due to survey fatigue may be encouraged to stay the course if the end of the study “draws visibly nearer with every question answered” (Heerwegh & Loosveldt, 2006, p. 194), progress bars can also benefit researchers. However, progress bars can also *reduce* study completion rates by de-motivating and discouraging participants. This is particularly the case if they suggest that more of a survey remains than is actually the case (Biffignandi & Bethlehem, 2021; Crawford et al., 2001); a problem may be exacerbated in surveys that are long or use skip logic (Müller et al., 2014).

Liu and Wronski (2018) recently found that completion rates were highest when no progress bar was displayed. However, although the differences in their study were significant (as might be expected in a sample of > 25,000), they were not large. The completion rate was 87.5% when there was no progress bar, and 86.8% when a progress bar was included at the top of a page: If 400 people began a study, this would yield final samples that differed only by three persons. This may be a price worth paying if it lets Turkers (who Amazon consider legally equivalent to self-employed people or contractors; Gleibs, 2017) judge whether a task is worth completing. However, because built-in progress bars can be inaccurate, researchers should consider including additional or alternative markers of progress (e.g., explicit statements along the lines of “Part 2 of 4,” “You are about two-thirds of the way through the survey,” “There is one more set of questions before we collect your demographic details”). Some participants explicitly recommended combining these sorts of comments with efforts to be encouraging, stating, for example, “I love a little encouragement—as in ‘You're doing great—only a few more questions!’ It makes me feel like people really care about what I'm doing.”

#### Survey length and repetition

Long surveys can discourage participation, cause poorer completion rates among people who *do* participate, and elicit hastier, shorter, and more “uniform” responses to items placed later in the survey (Galesic & Bosnjak, 2009; Liu & Wronski, 2018; Marcus et al., 2007). In a recent study, the median ideal and maximum survey lengths reported by participants were 10 and 20 minutes, respectively (Revilla & Ochoa, 2017). Few participants in our study specified an upper limit on study length, but a significant number felt surveys should be shorter. Piloting a study (using the same pool of workers who will participate in the main study) to ensure the survey length falls in reasonable bounds (relative to the compensation offered) is, therefore, advisable.

Representing more than 9% of frustrations were remarks like “There are too many synonyms used in the questions sometimes…I feel I am answering the same question over and over again.” Annoyance at the repetitiveness of questions was, therefore, far more prevalent than was annoyance at the mere length of a survey. This suggests that it is not just how *much* of a participant’s time a survey takes that matters, but how well that participant’s time is *used*.

Researchers may take for granted that multi-item (MI) measures of constructs (which contribute to the repetitiveness that frustrates participants) are inherently superior to single-item (SI) measures. They may also believe using MI scales to be a prerequisite for placing articles in top journals for whom “measurement reliability of the coefficient alpha kind…[is] sacrosanct” (Drolet & Morrison, 2001, p. 196). However, although MI measures are often psychometrically superior to their SI counterparts (Diamantopoulos et al., 2012; Sarstedt & Wilczynski, 2009), they do have drawbacks that can be offset by using SI scales. Drolet and Morrison (2001) argue that MI scales add “little information over a one- or, at most, two-item scale” (p. 198), because as items are added participants are more apt to ignore differences between items. Moreover, the “repetitive and onerous” (Robinson, 2018, p. 742) nature of MI scales can prompt straight-lining (an undesirable form of responding to which SI instruments are less vulnerable) and reduce response rates. SI scales lessen participant fatigue, resulting in higher response rates that may compensate for psychometric shortcomings (Sarstedt & Wilczynski, 2009).

Participants in our study *were* willing to tolerate some repetition, but disliked being asked “the same question several times with one word difference each time where the words are synonyms.” Such phrasing hints at a significant problem. MI measures permit the easy calculation of reliability coefficients, and for the purpose of satisfying manuscript reviewers, the higher those coefficients are, the better. When researchers characterize an instrument as having a high level of reliability, however, it would often be more accurate to say that they are acknowledging “a high level of item redundancy wherein essentially the same item is repeated in several different ways” (Boyle, 1991, p. 291). Countering the “more is better” mentality ingrained in many researchers, when inter-item correlations or alpha reliabilities are *too* high, this may be evidence that a scale is “too narrow and too specific” (Boyle, 1991, p. 291) and fails to capture the full scope of the construct of interest. Many researchers were taught that a high reliability coefficient is necessary but not sufficient for scale validity. Far fewer, we suspect, (us included) learned that a high reliability coefficient may be a sign that a measure *lacks* validity.

These comments are intended neither as a screed against all MI instruments or a blanket endorsement of SI scales. Rather, we hope they will encourage researchers to reflect on whether MI or SI scales are more appropriate for their purposes (Diamantopoulos et al., 2012; Rossiter, 2002). However, if a good, SI measure is available, we recommend researchers use it and reviewers support this choice. For example, when Robins et al. (2001) compared their single-item self-esteem measure and the Rosenberg Self-Esteem scale, they found that “disattenuated correlations were near unity” and the scales shared “almost all of their reliable variance” (p. 426). In such cases, we see little reason to ask participants ten questions instead of one.

### Limitations and future directions

Our study has several limitations. First, it relies on self-report data. Although we believe participants answered our questions in good faith, their remarks should be taken in conjunction with empirical findings. For example, although participants indicated that they would put forth more effort or be more attentive if they were better compensated, findings on this matter have been mixed. This does not, however, negate the ethical imperative to offer fair levels of pay.

Second, although we asked participants to provide open-ended accounts of frustrations with Turking, this was incidental to the primary purpose of the survey in which they participated (which was fielded to collect demographic data that would allow the first author to invite participants meeting eligibility criteria to participate in a separate study). Further work investigating the experiences of crowdsource workers would, therefore, be valuable. Arguably, for example, the power differential in the status accorded requesters and workers, and the level of remuneration offered to Turkers (and other crowdsource workers), suggests that there is little prestige attached to Turking; Gleibs (2017) considers Turking to be something that yields “a low-paid service income” (p. 1336). As such, research informed by scholarship on “dirty work” (Ashforth & Kreiner, 1999) might illuminate how Turkers discursively construct their identities.

Third, it is unclear how well our findings generalize from Turkers to crowdsource workers on other platforms that differ in important ways. For example, Prolific has a UK-centric participant pool, an emphasis on academic research, and a particular ethos regarding the treatment of participants. Researchers might, therefore, study participants from other crowdsourcing populations to compare positive and negative user experiences across platforms.

Finally, we recommend that researchers analyze which measures most often appear in crowdsourced studies. Just as meta-scientists have reported on the proportion of studies in their field that are conducted via crowdsourcing, we encourage micro-level research that assesses, for instance, the proportion of studies that ask participants to complete the Positive and Negative Affect Schedule (PANAS) or Rosenberg Self-Esteem inventory. This work would expand meaningfully on Marder and Fritz’s (2015) journalistic inquiry regarding the questions most often encountered by “Super Turkers.”

### Conclusions

Crowdsourcing offers researchers in the social and behavioral science the opportunity to move beyond the college student samples that have been the mainstay of so much of our work. It also presents a new set of challenges for researchers to navigate. The Turkers we recruited were quite forthcoming about their experiences, and often seemed to appreciate having the chance to offer feedback on issues that researchers (including ourselves) may not have appreciated were so important to them. We hope that the present study may be helpful for colleagues as they seek to implement respondent-centered research practices when using crowdsourcing platforms such as MTurk. We close by reiterating Gleibs’ (2017) recommendation:

First and foremost we should understand MTurk workers (or other members of crowdsourcing platforms) not as “subjects” or anonymous workers who provide us with easily accessible data, but as active participants who make important contributions to our work and research in general. (p. 1338)

## Footnotes

1 Wessling et al. (2017), for instance, recommend prior to launching their substantiative study, researchers create a brief pre-screening questionnaire. In a way that does not “give the game away,” researchers can mix questions that allow them to gauge whether eligibility criteria are met with filler items, and then make the main study available only to participants who met these eligibility conditions, either by setting qualifications within MTurk, or by creating participant “whitelists” via third-party platforms such as CloudResearch.

2 These response options reflect an error in survey construction: 11–14 should have been 11–15, and 15–20 should have been 16–20.

3 The second author recently collected data from two samples via Prolific. Respondents from the first sample had completed an average of 480 studies (ever). Respondents in sample 2 had completed an average of 620 studies.

4 Participants answered further questions that were used as covariates in the subsequent focal study, which were introduced as “items that I hope will help me think about how best to design a study I hope to do in the future.”

5 In the interests of full disclosure, three participants who reviewed this study noted that although the survey was well intentioned, they felt underpaid because it took them twice as long as estimated to complete the HIT.

6 Kung et al. (2018) provide a helpful clarification of the differences between attention checks (ACs) and instructional manipulation checks (IMCs), considering the latter to be a specific type of the former. However, although researchers may differentiate between these terms, the participants in our study referred *exclusively* to attention checks.

7 This is a conservative estimate as it does not include any of the 10% of frustrations relating to the clarity and accuracy of HIT description: We did not micro-code these frustrations for specific areas of opacity or inaccuracy, but a significant portion of them addressed inaccurate estimates of how long a HIT would take to complete.

8 We are not suggesting here that researchers be obligated to accept and pay for work that shows every sign of having been performed by a bot or that clearly represents deception on the part of a respondent (e.g., as evidenced by repeated submissions or inconsistent reporting of demographic characteristics).
